# High-CHO diet increases post-exercise oxygen consumption after a supramaximal exercise bout

**DOI:** 10.1590/1414-431X20165656

**Published:** 2016-10-24

**Authors:** G.A. Ferreira, R. Bertuzzi, F.R. De-Oliveira, F.O. Pires, A.E. Lima-Silva

**Affiliations:** 1 Grupo de Pesquisa em Ciências do Esporte, Centro Acadêmico de Vitória, Universidade Federal de Pernambuco, Vitória de Santo Antão, PE, Brasil; 2 Grupo de Estudos em Desempenho Aeróbio da USP, Escola de Educação Física e Esporte, Universidade de São Paulo, São Paulo, SP, Brasil; 3 Núcleo de Estudos do Movimento Humano, Departamento de Educação Física, Universidade Federal de Lavras, Lavras, MG, Brasil; 4 Grupo de Estudos em Psico-fisiologia do Exercício, Escola de Artes, Ciências e Humanidades, Universidade de São Paulo, São Paulo, SP, Brasil

**Keywords:** Excess post-exercise oxygen consumption, EPOC, Supramaximal exercise, Energy expenditure, High-intensity exercise

## Abstract

We investigated if carbohydrate (CHO) availability could affect the excess post-exercise oxygen consumption (EPOC) after a single supramaximal exercise bout. Five physically active men cycled at 115% of peak oxygen uptake (V̇O_2 peak_) until exhaustion with low or high pre-exercise CHO availability. The endogenous CHO stores were manipulated by performing a glycogen-depletion exercise protocol 48 h before the trial, followed by 48 h consuming either a low- (10% CHO) or a high-CHO (80% CHO) diet regime. Compared to the low-CHO diet, the high-CHO diet increased time to exhaustion (3.0±0.6 min *vs* 4.4±0.6, respectively, P=0.01) and the total O_2_ consumption during the exercise (6.9±0.9 L and 11.3±2.1, respectively, P=0.01). This was accompanied by a higher EPOC magnitude (4.6±1.8 L *vs* 6.2±2.8, respectively, P=0.03) and a greater total O_2_ consumption throughout the session (exercise+recovery: 11.5±2.5 L *vs* 17.5±4.2, respectively, P=0.01). These results suggest that a single bout of supramaximal exercise performed with high CHO availability increases both exercise and post-exercise energy expenditure.

## Introduction

Exercise intensity is an important factor determining the excess post-exercise oxygen consumption (EPOC) (1). It has been described that there is a curvilinear relationship between EPOC magnitude (total O_2_ consumed during recovery) and exercise intensity (1). A larger EPOC [effect size (ES)=0.9] is observed after high-intensity exercise, compared to moderate-intensity ones, even when the total work performed is matched (2). The EPOC magnitude is also related to the exercise duration, in particular after a high-intensity, supramaximal exercise (SE) bout (3). For example, Bahr et al. (3) demonstrated that the EPOC magnitude was positively correlated to the exercise duration of an SE performed at 108% peak oxygen uptake (V̇O_2 peak_), once EPOC increased from 5.6 to 16.3 L (ES=6.3) as the exercise duration increased from 2 to 6 min. It is interesting to note that a single SE bout performed until exhaustion is considered a time-efficient alternative to increase post-exercise energy expenditure. This may have clinical applications because a single SE bout would increase post-exercise fat oxidation and insulin sensibility (4). Therefore, any manipulation that increases the time length of an SE bout will directly affect EPOC magnitude. This would be of practical relevance because a larger EPOC will increase post-exercise energy expenditure, which in turn could help individuals engaged in weight loss programs to reduce their body mass more rapidly and ameliorate their metabolic profile.

Regarding the main strategies to prolong SE duration, there are some evidences suggesting that high-carbohydrate (CHO) availability has an important role (ES ranging from 0.7 to 2.4) (5–7). We have previously demonstrated that the time to exhaustion during an SE was increased after 48 h under a high-CHO diet, when compared to an isocaloric, low-CHO diet (ES=2.4) (6). This improvement in exercise tolerance with a high-CHO diet might be associated with several mechanisms such as an attenuation of central fatigue, a better maintenance of excitation-contraction coupling, a maintenance of CHO oxidation, and a reduced exercise-induced strain (8). In this regard, it could be hypothesized that the longer endurance time during an SE after a high-CHO diet period could lead to a greater EPOC and to a higher total energy expenditure in a given exercise training session, which could potentiate the body weight loss and reestablish metabolic balance in individuals engaged in training programs for health promotion. Thus, the interplay between SE duration and diet on post-exercise metabolism is an important point to consider when making decisions about training prescription and establishing weight-loss approaches.

Therefore, we performed new analyses using a data set from a large project previously published (6) in order to determine the effects of a high-CHO diet on the EPOC after an SE bout performed until exhaustion. We hypothesized that the high-CHO diet would increase SE tolerance, thereby increasing EPOC compared to an isocaloric, low-CHO diet.

## Material and Methods

### Participants

The sample size calculation was based on the ES of a high-CHO diet on the SE duration and on the ES of SE duration on the EPOC, reported in Lima-Silva et al. (6) and Bahr et al. (3). The expected ESs from these studies were 2.4 and 4.0, respectively. Thus, using the lowest ES (2.4), the minimum sample size required in this study for an alpha of 0.05 and a beta of 0.80 was 4 participants. Therefore, after receiving verbal and written explanations, and signing an informed consent, 5 physically active males (age 31.0±7.7 years, height 180.2±4.3 cm, body mass 77.0±7.7 kg, body fat 13.3±2.9%, V̇O_2 peak_ 48.6±11.5 mL·kg^-1^·min^-1^) volunteered to participate in this study, which was approved by the Ethics Committee of Universidade de São Paulo, SP, Brazil.

### Preliminary test and SE familiarization

Participants completed an incremental test to exhaustion (starting at 50 W and increasing 20 W every 3 min) on a cycle ergometer (Ergo Fit 167, Ergo-FitGmbH & Co., Germany) to determine their first and second lactate thresholds (LT_1_ and LT_2_, respectively) and V̇O_2 peak_. Exhaustion was assumed when participants were unable to maintain a pedal cadence above 60 rpm. Blood micro-samples (25 μL) were obtained at each stage from the earlobe and immediately analyzed for blood lactate concentration (La) [YSI 1500 Sport, Yellow Springs Instruments, USA]. The V̇O_2_ was continuously measured breath-by-breath (Quark b2, Cosmed, Italy). The blood La was plotted as a function of the workload, and the LT_1_ and LT_2_ were identified by a 3-segment linear regression (9). The 3-segment linear regression fitting was done using an interactive process with two initially unknown intercepts calculated from every possible combination of intersections. The intercepts that best shared the curve in three linear segments were assumed when the highest R^2^ value and the lowest residual sum of squares were attained. The LT_1_ was therefore defined as the workload corresponding to an initial change in the rate of lactate accumulation in the blood, while LT_2_ was defined as the workload corresponding to the second change in the rate of lactate accumulation. The V̇O_2 peak_ was defined as the highest 30-s V̇O_2 peak_ average at the end of the test.

**Figure 1:**
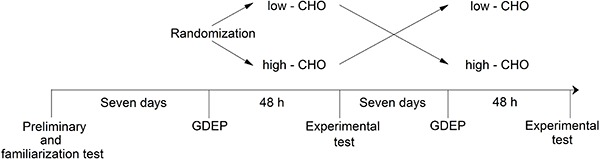
Experimental design. After the preliminary and familiarization test, and a 7-day period, participants were submitted to a glycogen-depletion exercise protocol (GDEP), followed by 48 h having either a high- or low- carbohydrate (CHO) diet. At the end of the 48-h period, participants returned to the laboratory and performed the test experiment for data collection. After a washout period of 7 days, the process was repeated with participants who had the high CHO diet previously, receiving the low CHO diet, and vice-versa.

Furthermore, in order to familiarize the participants with the experimental procedures, they performed an SE at 115% of V̇O_2 peak_ until exhaustion, 48 h after the incremental test (Figure 1). At this intensity (100 to 120% V̇O_2 peak_), the exercise can be tolerated for a sufficient time to develop a reasonable EPOC, as previously reported (3,10); time to exhaustion was also expected to be prolonged by the high-CHO diet in this exercise intensity, as reported elsewhere (6,7).

### Experimental tests

In order to produce a large difference in CHO availability, pre-SE endogenous CHO stores were altered by a combination of exercise and diet (11). First, participants performed a glycogen-depletion exercise protocol 48 h before each experimental session (12). This protocol consisted of a 90-min cycling at 50% of the difference between LT_1_ and LT_2_, followed by 6×1 min exercise bouts at 125% of V̇O_2 peak_; 1 min recovery was allowed between effort sets. After glycogen-depletion exercise protocol, participants followed an either high- or low- carbohydrate (CHO) diet for 48h. At the end of the 48-h period, participants returned to the laboratory and performed the test experiment for data collection. After a washout period of 7 days, the process was repeated with participants who had the high CHO diet previously, receiving the low CHO diet, and vice-versa (crossover design, Figure 1).

In the test day, participants arrived at 8:00 am in the laboratory after a 12-h overnight fast and rested on a chair during 20 min for the assessment of resting V̇O_2_ value (Quark b2, Cosmed, Italy). Then, they underwent a 5-min warm-up at 50 W, followed by an SE at 115% of V̇O_2 peak_ until exhaustion (6), which was assumed when participants were unable to maintain the pedal cadence above 60 rpm. Immediately after the test, they sat comfortably on a chair for 60 min. The V̇O_2 peak_ was measured continuously from the baseline to the end of the 60-min post-exercise period (Quark b2, Cosmed).

### Calculations

The rest V̇O_2 peak_ of the last 1 min of the resting period was averaged and used as baseline. The total O_2_ consumed during the exercise was calculated as the area under the V̇O_2_-time curve, after deduction of the corresponding V̇O_2_ rest (13). EPOC duration (min) was considered as the time taken for the V̇O_2_ to return to baseline values (when the mean of five consecutive 1-min V̇O_2_ values were within ±5% of the baseline V̇O_2_ (14). EPOC (L) was calculated as the integral of the V̇O_2_ versus time to reach the baseline minus the corresponding V̇O_2_ rest (15). All calculations were performed using the commercial software OriginPro 9.0 (OriginLab Corporation, USA). The total O_2_ required for the full session (exercise+recovery) was defined as the sum of the total O_2_ consumed during the exercise and EPOC. The total mechanical work performed was also calculated by multiplying external power output by exercise time duration.

### Statistical analysis

Smirnov-Kolmogorov test was used for testing normality. After confirmation of normal distribution, a paired *t*-test was used to compare the exercise duration, exercise O_2_ consumption, EPOC duration, EPOC magnitude, and total O_2_ consumption between low- and high-CHO diet. Cohen’s effect size was calculated and interpreted as proposed in (http://www.sportsci.org/resource/stats/): ≤0.2 = trivial, >0.2 and ≤0.6 = small, >0.6 and ≤1.2 = moderate, and >1.2 = large. The power effect for each analysis is also reported. The level of significance was set at P<0.05. All statistical procedures were performed with Statistic software version 10 (StataSoft Inc.¯, USA).

## Results

The maximal power output (PO) and V̇O_2 peak_ reached during the incremental test were 251.2±36.2 W and 3.7±0.6 L, respectively. The mean power output during the continuous exercise in the exercising-glycogen depletion protocol was 173.4±27.4 W (69.0±4.1% maximal power output during the incremental test). The supramaximal bouts of the exercising-glycogen depletion protocol (125% V̇O_2 peak_) were performed at 314.2±45.0 W. The SE was performed at 289.0±41.3 W.

**Figure 2:**
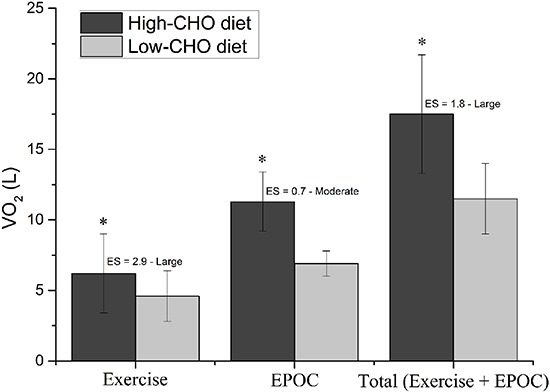
O_2_ consumption during exercise, post-exercise (excess post-exercise oxygen consumption, EPOC), and total oxygen demanded (exercise+recovery) in a supramaximal exercise bout performed until exhaustion, after pre-exercise carbohydrate (CHO) manipulation. ES: effect size. Data are reported as means±SD. *P<0.05 compared to low-CHO diet (paired *t*-test).

The baseline V̇O_2_ was similar between high- and low-CHO diet (0.34±0.6 *vs* 0.34±0.7 L/min, respectively, P=0.87, ES=0.05 trivial, power effect=0.05). There was a longer time to exhaustion (4.4±0.6 *vs* 3.0±0.6 min, P=0.01, ES=2.4 large, power effect=0.98), and greater total mechanical work (76.9±16.5 *vs* 50.9±9.4 kJ, P=0.001, ES=2.0 large, power effect=0.91) and total O_2_ consumed (11.3±2.1 *vs* 6.9±0.9 L, P=0.01, ES=2.9 large, power effect=0.99) in high- than in low-CHO diet (Figure 2). The V̇O_2_ measured at exhaustion was slightly higher in high- compared to low-CHO diet (48.6±11.0 and 45.2±11.0 mL·kg^-1^·min^-1^, respectively, P=0.004, ES=0.3 small, power effect=0.08).

The V̇O_2_ returned to baseline values within 60 min of rest in all participants and conditions. The EPOC duration was not significantly different between high- and low-CHO diet (30.0±12.4 and 21.0±15.2 min, respectively, P=0.16), although the ES was moderate (ES=0.7, power effect=0.27). However, the EPOC magnitude was significantly increased in high- than in low-CHO diet (6.2±2.8 and 4.6±1.8 L, respectively, P=0.03, ES=0.7 moderate, power effect=0.69, Figure 2). Consequently, the total O_2_ demanded (exercise+recovery) was greater in high- compared to low-CHO diet (P=0.01, ES=1.8 large, power effect=0.98, Figure 2).

## Discussion

In the present study, we investigated the effects of low- and high-CHO diet on total O_2_ consumed during and after a SE bout performed until exhaustion. The exercise tolerance was significantly longer in high- than in low-CHO diet, thereby inducing a higher VO_2_ during the exercise as well as after the exercise. These results suggest that high-CHO diet confers benefits considering exercise tolerance and energy expenditure during and after a single SE bout.

The high-CHO diet increased exercise duration (∼32%) and total mechanical work (∼34%) during a single SE bout. The increased tolerance further led to an increased exercise energy expenditure (i.e., ∼30% VO_2_ increase). Previous studies had already demonstrated that energy expenditure during a high-intensity exercise is proportional to its duration (16). Although the anaerobic metabolism is highly demanded during high-intensity exercises, the additional energy expenditure necessary to support longer SE with high-CHO availability is probably covered by aerobic metabolism (16,17). Although the mechanism for an increased SE tolerance with high-CHO availability is not fully understood, the assumption of elevated pre-exercise muscle glycogen stores seems to be improbable. Studies have shown that muscle glycogen is not fully depleted after a single SE bout performed until exhaustion (18), so that the remaining muscle glycogen would be sufficient to supply ATP and sustain a longer exercise. Therefore, fatigue during SE may be related to other factors such as a better metabolite balance, reduced exercise-induced strain and maintenance of excitation-contraction coupling (8). Additionally, the higher CHO availability can also play a central role by reducing the perceived effort during exercise, thus increasing the fatigue tolerance (8,19,20).

Another interesting result of this study was that the longer SE found with the high-CHO diet was accompanied by a greater EPOC magnitude, which indicates elevated post-exercise energy expenditure. The EPOC magnitude has been attributed to several mechanisms such as beta adrenergic stimulation, triglyceride/fatty acids cycling, body temperature, increased lactate removal and oxidation, replenishment of muscle and blood oxygen stores, and creatine phosphate resynthesis (1,21,22). Even though the shift from CHO to fat oxidation after the exercise bout with low-CHO availability may lead to an elevated EPOC (1,23), in the present study the EPOC magnitude was ∼34% greater with high-CHO diet. This result suggests that the exercise duration during an SE bout is an important factor influencing EPOC magnitude (1,14,24). Bahr et al. (3) demonstrated that EPOC increased 3 times (ES=6.3 large) with a 3 times longer SE (from 2 to 6 min). A similar proportion has also been demonstrated by Hagberg et al. (25 [Bibr B26] [Bibr B27]), who reported that EPOC increased 5 times when a high-intensity exercise (80% of V̇O_2 peak_) was 4 times longer (from 5 to 20 min). Interestingly, this ∼1:1 proportion and moderate to large ES persisted in the present study (i.e., 32% longer exercise, 34% larger EPOC magnitude, and large and moderate ES, respectively). In fact, longer exercise duration is thought to provoke larger metabolic disruption (1,3,22,), which might directly affect the return to homeostasis after the exercise. In this sense, prolonging the SE bout by ∼32% must have increased metabolic disturbance in the present study, and this may have been sufficient to proportionally increase EPOC magnitude (∼34%).

Because exercise and post-exercise VO_2_ were increased, the total energy expenditure demanded in the training session (exercise+recovery) was 34% larger in high- than in low-CHO diet. This result suggests that combining high-CHO diet and a SE bout until exhaustion could provide benefits considering energy expenditure. Recently, it has been debated that the lack of free time in modern life is a limiting factor for exercise practice and adherence (28–30). In this context, a short-duration SE bout until exhaustion has also been introduced as an alternative training model to stimulate active life style and to induce body weight reduction (4,30). In a practical perspective, a few minutes of SE per day could increase daily energy expenditure. Interestingly, a single SE bout until exhaustion has been suggested to elicit similar health benefits compared to several intermittent bouts of SE with short intervals for recovery, despite the shorter duration of the former (4). Thus, our results indicate that a high-CHO diet seems to confer additional benefits regarding the total energy expended during a SE bout performed until exhaustion. It would be interesting to investigate in further studies the accumulative effect of combining a high-CHO diet and training with a single exhaustive bout of SE on energy expenditure, weight loss and metabolic adaptations.

The main limitation of the present study is the small sample size. However, in an attempt to minimize possible bias, we calculated the Cohen’s ES between conditions for time to exhaustion, total O_2_ consumed, EPOC magnitude and total O_2_ demanded (exercise+recovery). Despite the small sample size, the statistical power ranged from 0.69 to 0.98 for total O_2_ consumed, EPOC magnitude and total O_2_ demanded (exercise+recovery). Therefore, the sample size was enough to achieve a satisfactory power effect, which in turn avoids a type II error. In addition, we are not able to extrapolate our results to a longitudinal perspective; further studies investigating the effect of combining a high-CHO diet and SE on energy expenditure and metabolic balance using a longitudinal-intervention design, are required.

In summary, when compared to a low-CHO diet, a high-CHO diet leads to a higher exercise tolerance, and higher consumed oxygen during and after a single exhaustive SE bout. From a practical standpoint, this might be an appealing strategy for a less time-consuming training and weight loss.
